# Assessing the potential of urinary dipstick testing to guide empirical antibiotic treatment for urinary tract infections in patients admitted to the emergency department: a cross-sectional study

**DOI:** 10.1017/ash.2026.10335

**Published:** 2026-04-07

**Authors:** Eugene Yevgeni Feigin, Roy Cohen, Moshe Giladi

**Affiliations:** 1 Gray Faculty of Medicine, Tel-Aviv University, Tel-Aviv, Israel; 2 Institute of Endocrinology, Metabolism and Hypertension, Tel-Aviv Sourasky University Medical Center, Tel-Aviv, Israel; 3 Internal Medicine Division, Tel-Aviv Sourasky University Medical Center, Tel-Aviv, Israel; 4 Edith Wolfson Medical Center, Holon, Israel

## Abstract

This study of 4,724 bacteriuria cases evaluated if urinary nitrites could guide empiric therapy. Nitrites lacked sensitivity and specificity, failing to accurately distinguish gram-positive from gram-negative pathogens. Because gram-positive infections correlate with age and comorbidities, clinicians should rely on patient characteristics rather than dipstick results to guide empiric antibiotic coverage.

## Introduction

Urinary tract infections (UTIs) are among the most common bacterial infections, typically managed with empiric antibiotics to provide rapid relief and prevent complications like sepsis.^1^ However, rising antimicrobial resistance and the overuse of broad-spectrum agents have led to frequent misalignment between empiric choices and actual pathogens.^2^

The urinary dipstick is a cost-effective point-of-care tool; combining leukocyte-esterase and nitrite tests yields a sensitivity of 75% and specificity of 82% for UTI diagnosis.^3^ Nitrites specifically indicate the presence of gram-negative (gram−) *Enterobacterales*, such as *E. coli.* ^4^ Consequently, current guidelines suggest that a positive nitrite test combined with clinical symptoms justifies empiric therapy targeting *E. coli* without further confirmation.^1^

However, standard empiric regimens may overlook gram-positive (gram+) pathogens, which are increasingly prevalent. *Enterococcus* species, now accounting for 8%–35% of UTIs, are particularly challenging due to inherent resistance to cephalosporins and aminoglycosides^5^. Notably, patients with *E. faecalis* UTIs are significantly more likely to receive inappropriate empirical therapy.^6^

Since gram+ bacteria do not produce nitrites, nitrite-negative pyuria may serve as a clinical indicator to shift empiric coverage.^7^

## Materials and methods

### Ethics approval

The Tel Aviv Sourasky Medical Center institutional review board (IRB) approved this study (TLV-0205-24).

### Inclusion criteria

Adult patients presented to the Department of Emergency Medicine (ER) of Tel-Aviv Sourasky Medical Center, Israel, between 1.7.2008–31.7.2023, in whom urinary dipstick test and urine culture were performed.

### Microbiology

Positive urine cultures were defined as those who grew pathogenic bacteria. Normal skin flora and vaginal flora were regarded as contaminants.

### Statistical analysis

Categorical variables were described using frequency and percentage. Continuous variables were evaluated for normal distribution using histograms and Q-Q plots and reported as mean with standard deviation. Scale variables were reported as median and interquartile range (IQR). The χ^2^ test compared categorical variables. Mann-Whitney U test was used to compare continuous, not normally distributed variables. All statistical tests were two-tailed. *P* ≤ .05 was considered statistically significant. Statistical analyses were performed using SPSS (IBM SPSS Statistics for Windows, version 27, IBM Corp., Armonk, NY, USA, 2020).

## Results

During the study period, 4,724 patients had both a dipstick test and a positive urine culture with growth of pathogenic bacteria (Table 1). Majority of cases were caused by gram− bacteria, accounting for 4,429 (93.8%) of the positive cultures. The most common uropathogen was *e. coli* (71.4%), followed by *klebsiella pneumonia* (10%), and *e. faecalis* (3.8%). Of the gram+ bacteria, *e. faecalis* was the most common (61%) (Table 1).

**Table 1.** Organisms identified by urine cultures in the study cohort

Gram negative			Gram positive		
Organism	N	Fraction%	Organism	N	Fraction%
*Escherichia coli*	3,377	76.2	*Enterococcus faecalis*	179	61
*Klebsiella Pneumoniae*	476	10.7	*Staphylococcus aureus*	50	17
*Proteus Mirabillis*	178	4.0	*Streptococcus species*	39	13
*Pseudomonas Aeruginosa*	88	2.0	*Enterococcus faecium*	11	4
*Klebsiella Aerogenes*	70	1.6	Other	16	5
*Citerobacter koseri*	67	1.5	Total	295	
*Enterobacter Cloacae*	44	1.0			
*Providencia Stuartii*	24	0.5			
*Klebsiella Oxytoca*	23	0.5			
*Acinetobacter Baumannii*	15	0.3			
*Serratia Marcescens*	11	0.2			
Other	56	1.3			
Total	4,429				

### Cohort characteristics

Out of 4,724 cases, 3,446 of the UTI cases occurred in females. The overall female predominance stems mainly from gram− pathogens. The F:M ratio in gram− UTIs is 2.9 while the F:M ratio is 1.1 for gram+ pathogens.

Patients with gram+ infections were older (62.9 ± 24.2 vs 56.2 ± 25.0 years, *P* < .001), had a higher CCI (4 [IQR 1,6] vs 3 [IQR 0,5], *P* = .049), higher creatinine (1.27 ± .94 vs 1.06 ± 0.77 mg/dL, *P* < .001), BUN (21.8 ± 19.3 vs 18 ± 16.8 mg/dL, *P* < .001), WBC (11.5 ± 4.9 vs 10 ± 5 10^9^ cells/L, *P* < .001), Neutrophil count (8.8 ± 4.6 vs 7.2 ± 4.2 10^9^ cells/L, *P* < .001). C-reactive protein (CRP) levels were similar in both groups (57.9 ± 71.5 vs 54.7 ± 69.3 mg/L, *P* = .594).

Positive leukocyte-esterase test had a high sensitivity of 85.8% (cut-off value ≥ 250 leukocytes/μL), compared with poor sensitivity of 41.4% for nitrites. The leukocyte esterase test had a higher sensitivity for gram− infections compared with gram+ infections (86.3% vs 76.9%, respectively, *P* < .001). Higher rates of nitrite positivity were observed for gram− infections, but nitrites were also detected in samples with the eventual growth of gram+ pathogens (44.4% vs 12.2%, *P* < .001).

### Sensitivity and specificity

Sensitivity and specificity analyses are shown in Table 2.

**Table 2.** Sensitivity and specificity analyses of urinary dipstick test results for predicting infections and their types

	All causes of UTI		Gram-positive UTI		Gram-negative UTI	
	SN%	SP%	SN%	SP%	SN%	SP%
**Nitrite (positive)**	41.4	89.0	12.2	81.0	44.4	89.0
**Leukocyte-esterase (positive)**	85.8	38.3	76.9	32.1	86.3	37.7
**Combinations**						
**Leukocyte-esterase (positive) nitrite (positive)**	36.0	92.5	9.8	84.8	38.7	92.5
**Leukocyte-esterase (positive) nitrite (negative)**	49.7	45.8	47.2	67.1	45.3	47.6
**Leukocyte-esterase (negative) nitrite (positive)**	5.4	96.5	2.4	95.9	5.7	96.5

Note. SN, sensitivity; SP, specificity.

While nitrites were not sensitive, their pooled specificity for UTI was high (89%). However, this was also true for gram+ infections (81%). A positive leukocyte esterase test was poorly specific overall (38.3%) regardless of the pathogen type (37.7% and 32.1% for gram− and gram+). Finally, we examined if any combination of the urinary dipstick test results could be used to discern between gram− and gram+ infections. Disappointingly, all the combinations lack sensitivity. The combination of positive leukocyte esterase and nitrite test has higher sensitivity for gram-negative infections (38.7% vs 9.8%), while positive leukocyte esterase and negative nitrite test has a higher specificity for gram-positive infections (67.1% vs 47.6%).

## Discussion

In our large cross-sectional study among adult patients, we found an overall prevalence of gram+ UTI of 6.2%, most of which were caused by *e. faecalis* (Table 1). However, gram-positive infections accounted for only 10.8% of nitrite-negative urine dipstick tests.

Strikingly, the presence of urinary nitrites did not exclude the presence of a gram-positive uropathogen, yielding a positive result in 12.2% of gram-positive UTIs.

UTIs are common and are usually diagnosed based on history, physical examination, and urinary dipstick test.^1^ Because most UTI treatment is empirical this is of special importance in the outpatient setting where urine cultures are not routinely obtained, or at the emergency department where the choice of antibiotic regiment is done quickly, and time to appropriate antibiotic treatment is closely associated with critically ill patients’ outcomes.^8^

Notably, inappropriate empiric antibiotic therapy was recently shown to result in increased risk for UTI-related hospitalization and re-consultation.^9^

Given the importance of appropriate empirical antibiotic therapy, previous studies addressed predictive factors for *e. faecalis* UTI.^6^ Consistent with our findings, the *e. faecalis* group had a higher proportion of men, older age, and more comorbidities. Disappointingly, the presence or absence of urinary nitrites lacks specificity and sensitivity for guiding empiric therapy.

Not only that relying on the absence of nitrites would lead to adding coverage for *e. faecalis* in ∼50% of gram-negative UTI cases, but we also show that nitrites may be present in cases caused by gram-positive organisms. Thus, the clinical presentation and patient characteristics, rather than the urinary dipstick test, should guide the choice of empiric antibiotic coverage.

There are several limitations to our study. First, it is limited by its retrospective design. Second, due to the locality of the cohort patient characteristics may largely vary in other places. Third, the case definition is complex and represents and inherent limitation.^10^ Due to the nature of retrospective cohort formation, the inclusion criterion were patients who presented to the ER in whom both a urine dipstick and a urine culture were performed. Although usually it is performed after a clinical decision by an ER physician or a nurse suggesting UTI was suspected, sometimes it is done as a workup of fever or sepsis without specific urinary complaints or when a patient is unable to pinpoint specific etiologies for their condition, affecting the rate of UTIs and asymptomatic bacteriuria in this large cohort. However, urinary colonization with bacteria, either in asymptomatic bacteriuria or urinary tract infections, that has nitrate reductase should still produce positive nitrites. Meaning that the production of nitrates and leukocyte esterase presence should be independent of symptoms and thus does not affect the validity of our conclusions.

In conclusion, nitrites or the lack of them are not necessarily correlated with infections and the pathogens causing them, and dipstick testing alone is not suitable for guiding empirical antibiotic coverage for suspected UTI.
